# Exploring Sexual Abuse, Adverse Childhood Experiences and Alcohol Use among Asian American Adults

**DOI:** 10.1007/s40653-026-00888-1

**Published:** 2026-04-29

**Authors:** Kruti S. Chaliawala, Rebecca A. Vidourek, Keith A. King

**Affiliations:** 1 School of Public and Population Health, Boise State University, Boise, ID USA; 2 Health Promotion and Education, Center for Prevention Science, University of Cincinnati, Cincinnati, OH USA

**Keywords:** Adverse childhood experiences, Sexual abuse, Alcohol use, Asian American adults, BRFSS

## Abstract

Childhood sexual abuse is prevalent across various communities of different socio-demographic backgrounds. Although existing literature on adverse childhood experiences and childhood sexual abuse tends to focus on various races across the spectrum, Asian American adults are often underrepresented due to the cultural stigma associated with sexual abuse. The current study aims to investigate childhood sexual abuse, adverse childhood experiences, and alcohol use among Asian American adults belonging to different income and education levels. Participants and Setting: The current study used a secondary analysis of the 2021 Behavioral Risk Factor Surveillance System (BRFSS) survey conducted by the CDC. In 2021, BRFSS interviewed 438,693 individuals via phone. Of these, *N* = 12,546 individuals self-identified as Asians for the “*preferred race*.” The descriptives suggest that of the *N* = 12,546 Asian American adults (5.9%, *n* = 738) reported experiencing sexual abuse, and (94.1%, *n =* 11,808) either refused to answer the question or did not know the answer. Furthermore, linear and bivariate regression analyses were conducted to understand the relationship between variables. The evidence of significant associations between CSA, ACEs, and any recent alcohol use is consistent with the idea that cultural stigma, family norms, gendered expectations, and individual coping strategies may influence both the occurrence and reporting of CSA. Understanding and addressing these factors are essential for promoting the overall health and well-being of Asian American communities.

## Introduction

As per the Centers for Disease Control (CDC, 2024), one in three girls and one in 13 boys in the United States experience childhood sexual abuse [CSA]; CSA is a grave public health concern as it encompasses various facets such as social, legal, and psychological dimensions of an individual’s well-being with the most detrimental effects on physical and psychological dimensions (Behere et al., 2013). Although sexual abuse is prevalent among all socio-economic and ethnic groups, various factors such as age, gender, minority, and disabilities increase the risk of CSA (Hines, 2007; Fassler et al., 2005; Putnam, 2003; Najman et al., 2005; Loeb et al., 2002). According to the US Department of Health and Human Services (2013), 21.8% Hispanic, 21% African American, 10.8% multiracial, 8.2% White, 4.7% Pacific Islander, 1.2% American Indian/Alaskan Native, 0.8% Asian American children have been exposed to abuse. These statistics reveal a distressing prevalence of CSA across different communities. However, it is crucial to note that CSA is the most under-reported abuse due to the stigma associated with various cultures (Gray & Rarick, 2018). Most racial differences examined through adult surveys have yielded the same results (Berliner, 2011; Fontes & Plummer, 2010; Jones et al., 2013; Lee et al., 2012; Olafson, 2011; Thompson et al., 2012).

Asian Americans represent a diverse and heterogeneous group within the United States, comprising individuals with diverse cultural, linguistic, and ethnic backgrounds. The term “Asian American” encompasses a vast population originating from East Asia (e.g., China, Japan, Korea), Southeast Asia (e.g., Vietnam, the Philippines, Thailand), and South Asia (e.g., India, Pakistan, Bangladesh), among other regions. These groups differ significantly in language, religion, migration history, socioeconomic status, and cultural norms (Cheung & LaChapelle, 2011; Lee & Choi, 2018). Although the U.S. Census and large-scale datasets, such as BRFSS, aggregate these subgroups under a single racial label, this practice risks masking critical differences in health behaviors, risk exposures, and help-seeking patterns (Fontes & Plummer, 2010; Hahm et al., 2012). The experiences, challenges, and strengths of Asian Americans are shaped by a complex interplay of factors, including immigration history, generational status, acculturation, and sociocultural influences. One key aspect of Asian American experiences is the concept of “model minority,” which emerged in the 1960s (Cheung & LaChapelle, 2011; Child Welfare Information Gateway [CWIG], 2016; Lee & Choi, 2018; Nadeem et al., 2020). This stereotype portrays Asian Americans as highly successful, academically gifted, and economically prosperous, often contrasting them with other minority groups. However, the model minority stereotype overlooks the diversity and disparities within the Asian American community, obscuring the challenges and inequalities faced by specific subgroups (Cheung & LaChapelle, 2011; CWIG, 2016; Lee & Choi, 2018; Nadeem et al., 2020). Asian Americans face various challenges and stressors impacting their mental health and well-being. Acculturative stress, which arises from adapting to a new culture while preserving one’s cultural heritage, can lead to psychological distress (Yoon et al., 2013). Language barriers, discrimination, and cultural clashes can also contribute to feelings of marginalization and identity conflicts (Nadal et al., 2014).

Adverse Childhood Experiences (ACEs) have been extensively studied in various populations and their impact on Asian American adults. Several studies have explored the relationship between childhood maltreatment and adverse outcomes in Asian American populations (Hahm et al., 2012; Lee & Choi, 2018). These studies underscore the importance of understanding the specific cultural factors and contexts that influence the experiences and consequences of childhood maltreatment in the Asian American population. Although studies have examined ACEs in different cultural settings (Nadeem et al., 2020), the intricate interplay between culture, family environment, and the severity of abuse remains a significant factor in predicting long-term outcomes (Fassler et al., 2005).

Within childhood sexual abuse (CSA) and adverse childhood experiences (ACEs), cultural factors can influence the prevalence, disclosure, and treatment of such incidents within Asian American communities. Cultural norms emphasizing collectivism, filial piety, and maintaining family harmony may create barriers to disclosure and seeking help for survivors of CSA (Futa et al. 2001a, b; Gilligan and Akhtar 2006). The stigma surrounding mental health issues and the preference for informal support networks may impede survivors’ access to appropriate services ( Ting & Hwang, 2009). Asian culture often shares conservative views regarding sexual abuse and adverse childhood experiences due to shame, guilt, denial, and respect for elders (Nadeem et al., 2020). As of 2014, Asian American children were the most underrepresented CSA victims in the child welfare system (Cheung & LaChapelle, 2011; CWIG, 2016). The paucity of literature regarding childhood sexual abuse and Asian American adults suggests a study focusing on this minority population (Robertson et al., 2016).

Cultural stigma surrounding childhood sexual abuse (CSA) varies across Asian subgroups. For example, South Asian communities may emphasize family honor and modesty, while East Asian cultures may prioritize filial piety and emotional restraint, both contributing to silence around abuse (Futa et al. 2001a, b; Gilligan and Akhtar 2006; Nadeem et al. 2020). These norms can inhibit disclosure, especially in phone-based surveys, where respondents may fear judgment or lack trust in confidentiality. Additionally, limited awareness of CSA definitions and formal support services further reduces response rates (Hollen, 2004; Robertson et al., 2016). Other ACEs, such as household mental illness, substance use, or parental separation, may also be interpreted differently across subgroups. For instance, some Southeast Asian families may normalize intergenerational trauma stemming from war or displacement, while South Asian families may conceal mental illness due to stigma. These cultural lenses shape both the experience and reporting of adversity (Jirapramukpitak et al., 2005; Lee & Choi, 2018).

The specific factors in reporting CSA may differ between Western and Asian cultures. The lower prevalence of CSA among Asian samples compared to non-Asian samples might be due to cultural and sexual norms and constraints in expressions among Asian cultures (Elliot & Urquiza, 2006). A study in Great Britain found that disclosure of CSA among Asians is often impeded by a lack of knowledge about CSA, lack of awareness regarding CSA services, fear of exposure, fear of culturally inappropriate responses by the professionals, and other cultural concepts such as shame, honor, and modesty (Hollen, 2004; Gilligan & Akhtar, 2006).

Moreover, alcohol use and alcohol-related disorders can present specific challenges for Asian Americans. Cultural values related to alcohol consumption, such as stigma or social reputation, can influence drinking behaviors and perceptions of alcohol-related problems (Chae et al. 2008a, b). Additionally, genetic factors affecting alcohol metabolism, such as the ALDH2*2 variant prevalent in East Asians, can increase the risk of adverse reactions to alcohol (Wall et al., 2012). Alcohol abuse is a coping mechanism observed among the survivors of ACEs and sexual abuse (Hines, 2007; Jones et al., 2013; Loudermilk et al., 2018). Furthermore, alcohol use has been identified as a potential consequence of ACEs and CSA (Chae et al. 2008a, b; Cook et al. 2021). Asian Americans, including those with a history of ACEs and CSA, may face specific challenges related to alcohol use and associated disorders (Chae et al. 2008a, b; Greene and Maggs 2020). Understanding the relationship between CSA, ACEs, and alcohol use among Asian adults in the US is crucial for developing effective prevention and intervention strategies (Child Welfare Information Gateway, 2016; Lee & Choi, 2018).

The current study explored sexual abuse as a category of Adverse Childhood Experiences (ACEs) among Asian adults using the 2021 BRFSS dataset. Furthermore, the association between demographic characteristics and 30-day alcohol consumption is reported. The research questions examined included:

1. What is the prevalence of sexual abuse among Asian American adults?
2. Does the sexual abuse reported within this population differ based on the number of adverse childhood experiences (ACEs) and demographic characteristics such as sex, education level, or income level?
3. Does the sexual abuse and the total number of ACEs experienced predict past 30-day alcohol consumption among Asian American adults?

## Methods

### Study Design

The current study used a secondary analysis of the 2021 Behavioral Risk Factor Surveillance System (BRFSS) data. The secondary data analysis was conducted upon approval from IRB. Specifically, the authors sought the Determination of Non-Human Subject research due to anonymity and public availability from the corresponding university IRB (2022 − 0805).

### Participants

In 2021, BRFSS interviewed 438,693 individuals via phone. Of these, *N* = 12,546 individuals self-identified as Asians for the “*preferred race*.” The questions regarding CSA and Adverse Childhood Experiences (ACEs) were optional, and the interviewer prompted at the beginning of the ACEs section that these questions could be skipped without prejudice.

### Procedure

The Behavioral Risk Factor Surveillance System (BRFSS) is a collaborative project between the states in the United States (US) and the Centers for Disease Control and Prevention (CDC). The BRFSS is a system of health-related telephone surveys designed to collect data on health-related risk behaviors, chronic health conditions, and the use of preventative services from the non-institutionalized adult population residing within the US (BRFSS, 2022). CDC’s Population Health Surveillance Branch administers the BRFSS under the Division of Population Health at the CDC’s National Center for Chronic Disease Prevention and Health Promotion.

In 2021, 54 states or territories used Computer-Assisted Telephone Interview (CATI) systems. Following the guidelines provided by BRFSS, state health personnel or contractors conduct interviews. The core portion of the questionnaire lasts about 17 min. Interview time for modules and state-added questions depends upon the questions used, which may add 5–10 min to the interview (BRFSS, 2022).

### Instrument

The current study utilized various variables from BRFSS to assess alcohol consumption, ACEs, and socio-demographics, such as income level, education level, and sex among Asian American adults. The respective questions for each variable are discussed below in their designated sections.

#### Adverse Childhood Experiences (ACEs)

Participants were asked the following questions regarding ACEs. Sexual abuse questions are included in ACEs. (1) Did you live with anyone who was depressed, mentally ill, or suicidal? (yes, no, don’t know); (2) Did you live with anyone who was a problem drinker or alcoholic? (yes, no, don’t know); (3) Did you live with anyone who used illegal street drugs or abused prescription medications? (yes, no, don’t know); (4) Did you live with anyone who served time or was sentences to serve time in a prison, jail or other correctional facility? (yes, no, don’t know); (5) Were your parents separated or divorced? (yes, no, parents not married); (6) How often did your parents or adults in home ever slap, hit, kick, punch or beat each other up? (never, once, more than once); (7) Not including spanking (before age 18), how often did a parent or adult in your home ever hit, beat, kick or physically hurt you in any way? (never, once, more than once); (8) How did a parent or adult in your home ever swear at you, insult you or put you down? (never, once, more than once); (9) How often did anyone at least five years older than you or an adult ever touch you sexually? (never, once, more than once); (10) How often did anyone at least five years older than you or an adult tried to make you touch them sexually? (never, once, more than once); and 11) How often did anyone at least five years older than you or an adult force you to have sex? (never, once, more than once).

#### Alcohol Consumption

The current study analyzed the following question regarding alcohol consumption. 1) During the past 30 days, did you have at least one drink of any alcoholic beverage such as wine, beer, a malt beverage, or liquor? (yes, no). This measure captures the presence or absence of recent drinking, but does not assess frequency, quantity, binge episodes, alcohol-related problems, or symptoms of alcohol use disorder.

#### Socio-demographics

The questions analyzed regarding socio-demographic characteristics are (1) What was your sex at birth? Was it male or female? (male, female); (2) What is the highest grade or year of school you completed? (never attended school, grades 1 through 8, grades 9 through 11, grade 12 or GED, college 1 year to 3 years, college 4 years or more); (3) Is your annual household income from all sources: (less than $10,000, less than $15,000, less than $20,000, less than $25,000, less than $35,000, less than $50,000, less than $75,000, $75,000 or more); and (4) Which one of these groups would you say best represents your race? (White, Black, or African American, American Indian, Alaska Native, Asian American, Pacific Islander).

## Results

### Participants

The total sample size consisted of *N* = 438,693. Of these, 12,546 individuals self-identified as Asians for the “*preferred race*.” Of these, 53.1% (*n* = 6668) self-identified as males and females (46.9%, *n =* 5878). The participant’s ages ranged from 18 years to 80 years (*M* = 43.93, *SD* = 17.10). The average income level was $50,000 to $100,000, and the education level was graduation from college. The average number of ACEs based on 11 categories of ACEs reported by the participant was *M* = 14.04, *SD* = 1.91. Of the sample, individuals who reported consumption of alcohol in the past 30 days were (40.8%, *n* = 5114). The frequencies demonstrating different types of sex, alcohol use, income level, and education level are presented in Table 1.

**Table 1:** The table demonstrates frequencies for sex, income level, alcohol use, and education level variables

Variable	Frequency (*n*)	Percentage (%)
Sex		
Male	1950	47.6
Female	2159	52.4
Education Level		
Did not graduate high school	323	2.6
Graduated High School	2077	16.7
Attended College or Technical School	2362	19.0
Graduated from college	7691	61.8
Income Level		
Less than $15,000	467	4.8
15,000–25,000	760	7.9
25,000–35,000	981	10.1
35,000–50,000	1127	11.6
50,000–100,000	2848	29.4
100,000–200,000	2515	26.0
200,000 - more	983	10.2
Drink Any Alcohol in Past 30 Days		
Yes	5114	44.8
No	6309	55.2

### Adverse Childhood Experiences (ACEs)

Of the *N* = 12,546 Asian American adults, 5.9% (*n* = 738) reported experiencing childhood sexual abuse, whereas 94.1% (*n* = 11,808) either refused to answer or did not know how to respond to the sexual abuse questions. Among those who completed the ACEs module (approximately 35% of the subsample), 8.4% (*n* = 332), 3.5% (*n* = 399), and 15.9% (*n* = 623) reported household mental illness, problem drinking, or parental separation/divorce, respectively. Simultaneously, 13.3% (*n* = 516), 21.4% (*n* = 830), and 28.8% (*n* = 1113) reported exposure to physical or verbal abuse in the home.

### Statistical Inferences

#### What is the Prevalence of Sexual Abuse among Asian American Adults?

Of the *N* = 12,546 Asian American adults (5.9%, *n* = 738) reported experiencing sexual abuse, and (94.1%, *n =* 11,808) either refused to answer the question or did not know the answer. Of 738 (7.2%, *n =* 51), participants reported being touched inappropriately by someone older than them. Additionally, (6.2%, *n =* 44) individuals reported being forced to touch someone inappropriately, and (3.2%, *n =* 23) reported having sex with someone older than them. Moreover, a cumulative score of all three categories of sexual abuse was computed, resulting in (3.0%, *n* = 22) reporting at least one type of sexual abuse, (4.5%, *n* = 33) indicating two types of sexual abuse, and (5.8%, *n* = 43) informing all the three types of sexual abuse.

#### Does the Sexual Abuse Reported within this Population Differ Based on the Total Number of Adverse Childhood Experiences (ACEs) and Demographic Characteristics such as Sex, Education Level, or Income Level?

A cumulative score of sexual abuse categories and ACEs was computed. The cumulative ACEs score of 11 categories did not include sexual abuse categories to avoid repetition. A multiple regression analysis was conducted. The dependent variable was the cumulative score of sexual abuse, and the independent variables were the cumulative score of ACEs, gender, education level, and income level. All the variables were entered into the regression model at the same time. The overall regression model was significant ((F (520, 4) = 9.929, *p* = 0.000)) with an R^2^ of 0.071 suggesting a variance of 7.1%. Sexual abuse is predicted by -1.178–0.085(ACEs) + 0.193 (Sex) – 0.022 (Income) + 0.010 (Education). The sex and the ACEs are significant predictors at *p* < 0.00. Overall, the results suggest that the total number of ACEs, income, sex, and education level of the participants within the sample predicts sexual abuse.

#### Does the Sexual Abuse and the Total Number of ACEs Experienced Predict the 30-day Alcohol Consumption among Asian American adults?

A cumulative score of sexual abuse and the total number of ACEs was computed into a single variable to analyze the data using bivariate regression analysis. The dependent variable was the combined scores of sexual abuse and the total number of ACEs, whereas the independent variable was drinking any alcoholic beverage in the past 30 days. The regression model was significant (F (625, 1) = 4.87, *p* = 0.028)) with an R^2^ of 0.008, suggesting a variance of 0.8%. Sexual abuse and ACEs are predicted by 26.846–0.448(Alcohol). Alcohol significantly predicts sexual abuse and ACEs at *p* < 0.05.

## Discussion

The results of the present study emphasize the need to move beyond the model minority stereotype and recognize the disparities and unique issues faced by this subgroup. Acculturative stress, language barriers, discrimination, and cultural clashes are some of the challenges that can impact the mental health and well-being of Asian Americans (Chae et al. 2008a, b; Futa et al. 2001a, b; Hahm et al. 2012). Additionally, cultural factors influence the prevalence, disclosure, and treatment of childhood sexual abuse (CSA) and adverse childhood experiences (ACEs) within Asian American communities (Fassler et al., 2005; Fontes & Plummer, 2010; Jirapramukpitak et al., 2005).

The prevalence of CSA (5.9%) is observed among Asian American adults through the findings of this study. The numbers presented by the statistics may not match the actual occurrences due to Asian cultural values, which perceive discussing sexual abuse as shameful. The burden of keeping the family together might create high tolerance for CSA and hinder the disclosure of CSA by Asian American adults to avoid social stigma (Futa et al. 2001a, b; Hahm et al. 2012; Weil and Lee 2004). As per the findings of the present study, Asian American adults reported experiencing ACEs (*M* = 26.14, *SD =* 2.5) as suggested by previous studies (Lee & Choi, 2018; Hahm et al., 2012). The strikingly high proportion of Asian American respondents who refused or were unable to answer questions about CSA and other ACEs should not be viewed only as a methodological weakness, but also as a substantive finding. The BRFSS items ask about experiences that are deeply stigmatized in many Asian families, including sexual victimization, parental mental illness, substance use, and interparental violence. For some subgroups, cultural norms that emphasize family honor, filial piety, and emotional restraint may discourage open acknowledgment of abuse or family conflict, especially in a brief telephone survey conducted by a governmental or medical authority (Futa et al. 2001a, b; Gilligan and Akhtar 2006; Nadeem et al. 2020). For others, immigration-related concerns, mistrust of institutions, limited English proficiency, or lack of familiarity with the concept of “abuse” as framed in U.S. public health discourse may further reduce willingness to disclose (Hollen, 2004; Robertson et al., 2016). These dynamics likely vary across ethnic subgroups, generations, and migration histories, but they converge in the BRFSS dataset as a high rate of nonresponse.

The present study shed light on the significant long-term consequences of childhood maltreatment within the Asian American population, highlighting the psychological distress and increased vulnerability to adverse mental health outcomes and risky behaviors. As discussed previously, the impact of ACEs on Asian American adults is a critical area of concern, as it underscores the need for a deeper understanding of the cultural factors and contexts that influence the experiences and consequences of childhood maltreatment within these communities. Moreover, recognizing the complex interplay between culture, family environment, and the severity of abuse is essential in predicting and addressing the long-term outcomes (Fassler et al., 2005). This collective body of research underscores the urgency of developing tailored interventions and support systems to address the specific challenges and needs of Asian American adults who have experienced childhood maltreatment.

Alcohol use among Asian American adults may be affected by various factors. Asian American cultures often have distinct attitudes and beliefs regarding alcohol consumption. Some Asian cultures view alcohol as an integral part of social gatherings and celebrations, while others may have a more conservative approach toward alcohol use. These cultural norms can influence the patterns of alcohol consumption and the perception of alcohol-related problems within the community (Hahm et al., 2012). Another factor that may affect alcohol consumption is acculturative stress. Acculturative stress, combined with discrimination and language barriers, may lead to higher levels of alcohol consumption as a coping mechanism to deal with these stressors (; Lee et al., 2012). Asian Americans often face barriers to accessing appropriate alcohol use treatment and support services. These barriers include limited culturally competent services, language barriers, lack of awareness about available resources, and low help-seeking behaviors. These factors contribute to health disparities and hinder the early identification and intervention of alcohol-related problems (Yoo et al., 2018).

As suggested by the present study, alcohol use is prevalent among Asian American adults, with approximately 44% (*n* = 5114) reporting drinking any alcoholic beverage in the last 30 days. Previous research indicates that alcohol abuse has significantly increased among the Asian population (Chae et al. 2008a, b). Between 2001 and 2013, alcohol consumption among the Asian American population increased by 29.1% (Grant et al., 2017). Several researchers have shared their concern regarding the underrepresentation of Asian American adults in alcohol abuse, use, or disorder studies, which may contribute to long-term adversities within a population susceptible to cardiovascular disease from alcohol consumption (Cook et al., 2021; Greene & Maggs, 2020). Alcohol consumption is considered a coping mechanism amongst ACEs and CSA survivors, and the current study reconfirms the same.

Although the number of Asian American respondents who completed the ACEs and CSA items was modest relative to the full BRFSS sample, and nonresponse to these sensitive questions was high, the findings remain meaningful for several reasons. First, even within this subset, hundreds of Asian American adults reported exposure to CSA and other ACEs, providing rare, population-based estimates for a group that is typically either numerically underrepresented or aggregated into an “Other” category in large datasets. Second, the very high proportion of refusals and “don’t know” responses is itself informative: it reflects the depth of stigma, silence, and cultural taboos surrounding sexual victimization, mental illness, and family conflict in many Asian communities, and supports the argument that CSA in Asian American populations is likely underrecognized and underreported rather than rare. Third, despite the modest explained variance in alcohol use, the observed associations between CSA, cumulative ACEs, and drinking suggest that when Asian American adults do report childhood victimization and adversity, these experiences are linked to their current alcohol consumption. Taken together, these points indicate that the present study should be interpreted as an important initial step toward documenting hidden adversity among Asian American adults, rather than as a definitive estimate of prevalence.

### Implications

Understanding the diverse needs and experiences of Asian Americans has significant public health implications. Policymakers, healthcare professionals, and community organizations must promote culturally appropriate prevention efforts, increase access to mental health services, and foster supportive environments (Child Welfare Information Gateway, 2016; Hahm et al., 2012; Weil & Lee, 2004).

To address CSA and ACEs, public health initiatives should focus on raising awareness, promoting early identification, and providing culturally sensitive support services. This includes enhancing community education programs, training healthcare providers to recognize and respond to signs of abuse and establishing support networks that are sensitive to the cultural norms and values of Asian American communities (Berliner 2011; Futa et al. 2001a, b; Robertson et al. 2016). Regarding alcohol use among Asian Americans, it is crucial to address cultural beliefs and attitudes toward alcohol consumption. Recognizing the influence of cultural factors and genetic predispositions to alcohol-related conditions, public health interventions should emphasize prevention, education, and targeted interventions. These efforts should address the social and environmental determinants of alcohol misuse and promote healthier coping mechanisms and stress management strategies (Cook et al., 2021; Greene & Maggs, 2020).

### Recommendations

Public health authorities should proactively devise and implement preventive strategies considering the cultural norms, linguistic preferences, and cultural nuances of various Asian American communities. It is imperative to develop targeted interventions that specifically address the unique challenges faced by different Asian American populations (Hahm et al., 2012). For those within these communities who have endured childhood sexual abuse (CSA), adverse childhood experiences (ACEs), or are at risk of alcohol use, efforts should be made to enhance access to culturally sensitive counseling and therapy services. This initiative should encompass the education of health professionals about the cultural factors that impact the disclosure and management of these issues (CWIG, 2016).

Furthermore, establishing community support groups and safe havens for survivors of CSA and ACEs, as well as individuals grappling with alcohol use, should be prioritized (Berliner, 2011; Robertson et al., 2016). These initiatives may serve as a refuge, contributing to the breaking of silence and dismantling the stigma surrounding these issues (Berliner, 2011; Robertson et al., 2016). Additionally, equipping social service workers, educators, and healthcare professionals with cultural competency training is crucial. Such training enhances their understanding of the cultural factors that influence the experiences and needs of Asian Americans (Cook et al., 2021).

### Limitation

Several limitations should be considered when interpreting these findings. First, this study relies on secondary analysis of the 2021 Behavioral Risk Factor Surveillance System (BRFSS), which constrains the analysis to the original survey’s design, including its optional Adverse Childhood Experiences (ACEs) and childhood sexual abuse (CSA) modules. The phone-based format and optional nature of these items likely contributed to high non-response rates, particularly among Asian American participants; only 5.9% (*n* = 738) reported CSA, while 94.1% (*n* = 11,808) either refused or responded “don’t know,” suggesting substantial underreporting and limiting prevalence accuracy (BRFSS, 2022). Second, the reliance on retrospective self-reports introduces potential recall and mood-related biases, as adults may misremember or reinterpret childhood experiences (Gilbert et al. 2009). While common in ACEs research (Crouch et al., , 2018; Loudermilk et al., 2018; Windle et al., 2018), this method has inherent limitations. Additionally, phone surveys may exclude individuals with limited English proficiency or unreliable phone access, potentially underrepresenting certain Asian American subgroups (BRFSS, 2022). Third, cultural stigma surrounding sexual victimization in many Asian communities is linked to family honor, filial piety, and fear of “losing face,” which may suppress disclosure (Futa et al. 2001a, b; Gilligan and Akhtar 2006; Nadeem et al. 2020). Survivors may also lack awareness of CSA or fear culturally insensitive responses (Hollen, 2004; Robertson et al., 2016), contributing to the high rate of missing data and likely underestimating CSA prevalence. Fourth, the regression models explained limited variance in outcomes (*R*² = 0.071 for CSA; *R*² = 0.008 for alcohol use), indicating that other factors such as acculturative stress, discrimination, mental health, and social norms likely influence alcohol use (Chae et al. 2008a, b; Greene and Maggs 2020; Hahm et al. 2012). Moreover, alcohol use was measured using a single yes/no item, which limited the ability to assess frequency, quantity, or problematic patterns.

Fifth, the BRFSS categorizes all Asian Americans under a single racial label, masking heterogeneity across ethnic subgroups (Cheung & LaChapelle, 2011; Lee & Choi, 2018). Without disaggregated data, the study could not examine subgroup-specific patterns in CSA, ACEs, or alcohol use, despite evidence that such differences exist (Fassler et al., 2005; Fontes & Plummer, 2010; Hahm et al., 2012). Finally, the cross-sectional design precludes causal inference. Although CSA and ACEs occurred in childhood and alcohol use reflects recent behavior, all variables were measured simultaneously, limiting the ability to determine temporal or directional relationships (Arata et al., 2007; Peltzer & Pengpid, 2016; Tran et al., 2015). Longitudinal studies are needed to clarify these pathways.

## Conclusion

The findings from this study suggest that childhood sexual abuse (CSA) and adverse childhood experiences (ACEs) are reported by a subset of Asian American adults in the BRFSS dataset, and that these experiences are modestly associated with recent alcohol use. Although the overall prevalence of CSA was low (5.9%), the exceptionally high rate of non-response (94.1%) to CSA items indicates potential cultural barriers to disclosure. Regression analyses showed that CSA and cumulative ACEs were statistically significant predictors of past 30-day alcohol use. However, the explained variance was slight (*R*² = 0.008), suggesting that other unmeasured factors also contribute to alcohol-related outcomes. These patterns of limited disclosure, modest associations, and high non-response rates highlight the need for more culturally sensitive research tools and disaggregated data based on subgroups to better understand adversity among Asian American populations. Although this study does not establish causality or fully capture the complexity of cultural, social, and individual influences, it provides initial population-level evidence that CSA and ACEs are present and potentially have an impact on this underrepresented group. Future research should build on these findings with longitudinal designs and culturally tailored methodologies.

## Data Availability

Not Applicable.

## References

[CR1] Arata, C. M., Langhinrichsen-Rohling, J., Bowers, D., & O’Brien, N. (2007). Differential correlates of multi-type maltreatment among urban youth. *Child Abuse and Neglect*, *31*, 393–415. 10.1016/j.chiabu.2006.09.006 17412420

[CR2] Behere, P. B., Rao, S., & Mulmule, T. S., A. N (2013). Sexual abuse in women with special reference to children: *Barriers, boundaries and beyond*. *Indian Journal of Psychiatry*, *55*, 316–319. 24459299 10.4103/0019-5545.120535 PMC3890930

[CR3] Berliner, L. (2011). Child sexual abuse: Definitions, prevalence, and consequences. In E. B. Myers (Ed.), *The APSCA Handbook on Child Maltreatment* (Vol. 3, pp. 215–232). Sage.

[CR47] Centers for Disease Control and Prevention. (2023). Behavioral Risk Factor Surveillance System: 2022 summary data and documentation https://www.cdc.gov/brfss/annual_data/annual_2022.html

[CR45] Centers for Disease Control and Prevention. (2024). About child sexual abuse https://www.cdc.gov/child-abuse-neglect/about/about-child-sexual-abuse.html

[CR4] Chae, D. H., Gavin, A. R., & Takeuchi, D. T. (2008a). Smoking prevalence among Asian Americans: Findings from the National Latino and Asian American Study (NLAAS). *Public Health Reports*, *123*(6), 769–776. 10.1177/003335490612100616 PMC1781917 17278411

[CR5] Chae, D. H., Takeuchi, D. T., Barbeau, E. M., Bennett, G. G., Lindsey, J. C., Stoddard, A. M., & Krieger, N. (2008b). Alcohol disorders among Asian americans: Associations with unfair treatment, racial/ethnic discrimination, and ethnic identification (the national Latino and Asian Americans study, 2002–2003). *Journal of Epidemiology and Community Health (1979)*, *62*(11), 973–979. 10.1136/jech.2007.066811 18854501

[CR7] Cheung, M., & LaChapelle, A. (2011). Disproportionality from the other side: The underrepresentation of Asian American children. In D. K. Green, K. Belanger, R. G. McRoy, & L. Bullard (Eds.), *Challenging racial disproportionality in child welfare: Research, policy, and practice* (pp. 131–139). CWLA.

[CR8] Child Welfare Information Gateway. (2016). *Racial disproportionality and disparity in child welfare*. US Department of Health and Human Services, Children’s Bureau.

[CR9] Cook, W. K., Tam, C. C., Luczak, S. E., Kerr, W. C., Mulia, N., Lui, C., & Li, L. (2021). Alcohol consumption, Cardiovascular-Related conditions, and ALDH22 ethnic group prevalence in asian americans. *Alcoholism Clinical and Experimental Research*, *45*(2), 418–428. 10.1111/acer.14539 33349921 PMC8545194

[CR10] Crouch, E., Radcliff, E., Strompolis, M., & Wilson, A. (2018). Adverse childhood experiences (ACEs) and alcohol abuse among South Carolina adults. *Substance Use & Misuse*, *53*(7), 1212–1220. 10.1080/10826084.2017.1400568 29185846

[CR11] Elliott, K., & Urquiza, A. (2006). Ethnicity, culture and child maltreatment. *Journal of Social Issues*, *62*, 787–809. 10.1111/j.1540-4560.2006.00487.x

[CR12] Fassler, I. R., Amodeo, M., Griffin, M. L., Clay, C. M., & Ellis, M. A. (2005). Predicting long-term outcomes for women sexually abused in childhood: contribution of abuse severity versus family environment. *Child Abuse and Neglect*, *29*, 269–284. 15820543 10.1016/j.chiabu.2004.12.006

[CR13] Fontes, L. A., & Plummer, C. (2010). Cultural issues in disclosures of child sexual abuse. *Journal of Child Sexual Abuse*, *19*(5), 491–518. 10.1080/10538712.2010.512520 20924908

[CR14] Futa, K. T., Higa-McMillan, C., & Weiss, B. (2001a). Cultural factors influencing the mental health of Asian Americans. *Western Journal of Medicine*, *175*(5), 349–353. 12208826 PMC1071736

[CR15] Futa, K. T., Hsu, E., & Hansen, D. J. (2001b). Child sexual abuse in Asian American families: An examination of cultural factors that influence prevalence, identification, and treatment. *Clinical Psychology Science and Practice*, *8*, 189–209. 10.1093/clipsy.8.2.189

[CR49] Gilbert, R., Widom, C. S., Browne, K., Fergusson, D., Webb, E., & Janson, S. (2009). Burden and consequences of child maltreatment in high-income countries. *The Lancet*, *373*(9657), 68–81. 10.1016/S0140-6736-08-61706-7 19056114

[CR16] Gilligan, T. D., & Akhtar, S. (2006). Sexual trauma in Asian-American women: A cultural analysis. *Psychoanalytic Psychology*, *23*(2), 334–347.

[CR17] Grant, B. F., Chou, S. P., Saha, T. D., Pickering, R. P., Kerridge, B. T., Ruan, W. J., Huang, B., et al. (2017). Prevalence of 12- month alcohol use, high-risk drinking, and DSM-IV alcohol use disorder in the United States, 2001–2002 to 2012–2013: results from the National Epidemiologic Survey on Alcohol and Related Conditions. *JAMA Psychiatry*, *74*, 911–923. 28793133 10.1001/jamapsychiatry.2017.2161 PMC5710229

[CR46] Gray, S., & Rarick, S. (2018). Exploring gender and racial/ethnic differences in the effects of child sexual abuse. *Journal of Child Sexual Abuse*, *27*(5), 570–587. 10.1080/10538712.2018.1484403 29924694

[CR18] Greene, K. M., & Maggs, J. L. (2020). Longitudinal change in alcohol use and motivations for drinking among asian american college students. *Alcoholism Clinical and Experimental Research*, *44*(10), 2109–2117. 10.1111/acer.14436 33460235 PMC7815956

[CR19] Hahm, H. C., Kolaczyk, E., Lee, Y., Jang, J., & Ng, L. (2012). Do Asian-American women who were maltreated as children have a higher likelihood for HIV risk behaviors and adverse mental health outcomes? *Women’s Health Issues*, *22*, e35–e43. 10.1016/j.whi.2011.07.003 21872488 PMC3236805

[CR20] Hines, D. A. (2007). Predictors of sexual coercion against women and men: a multilevel, multinational study of university students. *Archives of sexual behavior*, *36*(3), 403–422. 10.1007/s10508-006-9141-4 17333324

[CR21] Hollen, C. V. (2004). HIV/AIDS and the gendering stigma in Tamil Nadu, South India. *Culture Medicine and Psychiatry*, *34*(4), 633–657. 10.1007/s11013-010-9192-9 20842521

[CR22] Jirapramukpitak, T., Prince, M., & Harpham, T. (2005). The experience of abuse and mental health in the young Thai population: A preliminary survey. *Social Psychiatry and Psychiatric Epidemiology*, *40*, 955–963. 10.1007/s00127-005-0983-1 16328752 PMC1800824

[CR23] Jones, D. J., Lewis, T., Litrownik, A., Thompson, R., Proctor, L. J., Isbell, P., Dubowitz, H., English, D., Jones, B., Nagin, D., & Runyan, D. (2013). Linking childhood sexual abuse and early adolescent risk behavior: the intervening role of internalizing and externalizing problems. *Journal of Abnormal Child Psychology*, *41*(1), 139–150. 10.1007/s10802-012-9656-1 22752719 PMC3479370

[CR25] Lee, C., Cronley, C., White, H. R., Mun, E. Y., Stouthamer-Loeber, M., & Loeber, R. (2012). Racial differences in the consequences of childhood maltreatment for adolescent and young adult depression, heavy drinking, and violence. *Journal of Adolescent Health*, *50*(5), 443–449. 10.1016/j.jadohealth.2011.09.014 PMC3336090 22525106

[CR26] Lee, J., & Choi, M. J. (2018). Childhood shadows: Psychological distress of childhood maltreatment among Asian-American women. *Journal of Child and Family Studies*, *27*, 2954–2965. 10.1007/s10826-018-1114-4

[CR27] Loeb, T. B., Williams, J. K., Carmona, J. V., et al. (2002). Child sexual abuse: associations with the sexual functioning of adolescents and adults. *Annual Review of Sex Research*, *13*, 307–345. 12836735

[CR28] Loudermilk, E., Loudermilk, K., Obenauer, J., & Quinn, M. A. (2018). Impact of adverse childhood experiences (ACEs) on adult alcohol consumption behaviors. *Child Abuse & Neglect*, *86*, 368–374. 10.1016/j.chiabu.2018.08.006 30241703

[CR30] Nadal, K. L., Wong, Y., Griffin, K. E., Davidoff, K. C., & Sriken, J. (2014). The Filipino values scale: Construction, validation, and application. *Journal of Multicultural Counseling and Development*, *42*(4), 226–242.

[CR31] Nadeem, T., Asad, N., & Hamid, S. N. (2020). Cultural considerations in providing trauma care to female, childhood sexual abuse survivors: Experiences from Pakistan. *Asian Journal of Psychiatry*, *48*, 101885. 10.1016/j.ajp.2019.101885 31835141

[CR32] Najman, J. M., Dunne, M. P., Purdie, D. M., Boyle, F. M., & Coxeter, P. D. (2005). Sexual abuse in childhood and sexual dysfunction in adulthood: an Australian population-based study. *Archives of Sexual Behavior*, *34*(5), 517–526. 10.1007/s10508-005-6277-6 16211473

[CR33] Olafson, E. (2011). Child sexual abuse: Demography, impact, and interventions. *Journal Of Child & Adolescent Trauma*, *4*(1), 8–21. 10.1080/19361521.2011.545811

[CR34] Peltzer, K., & Pengpid, S. (2016). Childhood physical and sexual abuse and adult health risk behaviours among university students from 24 countries in Africa, the Americas, and Asia. *Journal of Psychology in Africa*, *26*, 149–155. 10.1080/14330237.2016.1163899

[CR35] Putnam, F. W. (2003). Ten-year research update review: Child sexual abuse. *Journal of the American Academy of Child and Adolescent Psychiatry*, *42*(3), 269–278. 10.1097/00004583-200303000-00006 12595779

[CR36] Robertson, H. A., Nagaraj, N. C., & Vyas, A. N. (2016). Family violence and child sexual abuse among South-Asians in the US. *Journal of Immigrant and Minority Health*, *18*, 921–927. 10.1007/s10903-015-0227-8 26032775

[CR37] Thompson, N. J., McGee, R. E., & Mays, D. (2012). Race, Ethnicity, Substance Use, and Unwanted Sexual Intercourse among Adolescent Females in the United States. *Western Journal of Emergency Medicine*, *13*(3), 283–288. 10.5811/westjem.2012.3.11774 22900127 PMC3415834

[CR38] Ting, J. Y., & Hwang, W. C. (2009). Cultural influences on help-seeking attitudes in Asian American students. *American Journal of Orthopsychiatry*, *79*(1), 125–132. 10.1037/a0015394 19290732

[CR39] Tran, Q. A., Dunne, M. P., Vo, T. V., & Luu, N. H. (2015). Adverse childhood experiences and the health of university students in eight provinces of Vietnam. *Asia-Pacific Journal of Public Health*, *27*(8, Suppl.), 26S–32S. 10.1177/1010539515589812 26047629

[CR40] US Department of Health and Human Services Administration for Children and Families, Administration on Children, Youth and Families, Children’s Bureau. (2013). *Child maltreatment 2012*. Retrieved from https://www.acf.hhs.gov/archive/cb/report/child-maltreatment-2013

[CR41] Wall, T. L., Shea, S. H., Chan, K. K., Carr, L. G., & Li, T. K. (2012). Ethnic differences in sensitivity to the depressant effects of alcohol. *Alcoholism: Clinical and Experimental Research*, *36*(6), 965–972.

[CR42] Weil, J. M., & Lee, H. H. (2004). Cultural considerations in understanding family violence among Asian American Pacific Islander families. *Journal of Community Health Nursing*, *21*, 217–227. 10.1207/s15327655jchn2104_2 15537547

[CR43] Windle, M., Haardörfer, R., Getachew, B., Shah, J., Payne, J., Pillai, D., & Berg, C. J. (2018). A multivariate analysis of adverse childhood experiences and health behaviors and outcomes among college students. *Journal of American College Health*, *66*(4), 246–251. 10.1080/07448481.2018.1431892 29405856 PMC5948167

[CR48] Yoo, J., & Miyamoto, Y. (2018). Cultural fit of emotions and health implications: A psychosocial resources model. *Social and Personality Psychology Compass*, 12 (2), e12372 10.1111/spc3.12372

[CR44] Yoon, E., Chang, C. T., Kim, S., Clawson, A., Cleary, S. E., Hansen, M., & Novilla, M. L. B. (2013). A meta-analysis of acculturation/enculturation and mental health. *Journal of Counseling Psychology*, *60*(1), 15–30. 23163612 10.1037/a0030652

